# The N-Pact Factor: Evaluating the Quality of Empirical Journals with Respect to Sample Size and Statistical Power

**DOI:** 10.1371/journal.pone.0109019

**Published:** 2014-10-08

**Authors:** R. Chris Fraley, Simine Vazire

**Affiliations:** 1 Department of Psychology, University of Illinois at Urbana-Champaign, Champaign, Illinois, United States of America; 2 Department of Psychology, University of California Davis, Davis, California, United States of America; Hellas, Greece

## Abstract

The authors evaluate the quality of research reported in major journals in social-personality psychology by ranking those journals with respect to their *N*-pact Factors (NF)—the statistical power of the empirical studies they publish to detect typical effect sizes. Power is a particularly important attribute for evaluating research quality because, relative to studies that have low power, studies that have high power are more likely to (a) to provide accurate estimates of effects, (b) to produce literatures with low false positive rates, and (c) to lead to replicable findings. The authors show that the average sample size in social-personality research is 104 and that the power to detect the typical effect size in the field is approximately 50%. Moreover, they show that there is considerable variation among journals in sample sizes and power of the studies they publish, with some journals consistently publishing higher power studies than others. The authors hope that these rankings will be of use to authors who are choosing where to submit their best work, provide hiring and promotion committees with a superior way of quantifying journal quality, and encourage competition among journals to improve their NF rankings.

## Introduction

Most researchers in psychology strive to publish their best work in highly regarded journals. Publishing in a top journal is considered a mark of accomplishment, and articles that appear in reputable journals are more likely to be recognized and cited by one's colleagues. Moreover, it is not uncommon for search and promotion committees to judge a researcher's work on the reputation of the journals in which he or she publishes.

But how do we know whether a given journal has a track record of publishing high-quality research? The most common way to quantify the quality of scientific journals in psychology is with respect to their citation *Impact Factors* (IF; such as the Thomson Reuters Journal Citation Reports)—an index of how often articles published in those journals are cited. Although impact factors are widely used in academia, their use is controversial [1]–[4]. Some critics, for example, have questioned whether the impact of research is appropriately indexed over a relatively short time span (i.e., the two years following publication) compared to longer time spans [5]. In addition, a number of critics have argued that citation rates *per se* may not reflect anything informative about the *quality* of empirical research. A paper can receive a large number of citations in the short run because it reports surprising, debatable, or counter-intuitive findings regardless of whether the research was conducted in a rigorous manner. In other words, the short-term citation rate of a journal may not be particularly informative concerning the quality of the research it reports.

The “quality” of a research study, however, can be an elusive thing to quantify. And, as scholars have demonstrated, different scientists evaluating the same manuscripts do not always agree on the quality of the work in question [6]. Thus, one challenge for the field is to develop useful ways to index the quality of published empirical research. Such indices would help researchers and promotion committees better evaluate various journals, allow the public and the press (i.e., consumers of scientific knowledge in psychology) to have a better appreciation of the credibility of published research, and perhaps even facilitate competition among journals in a way that would improve the net quality of published research.

One potentially valuable way to index the quality of empirical research is with respect to the statistical power of research designs to detect the average effect in a research area. *Statistical power* is defined as the probability of detecting an effect of interest when that effect actually exists [7]. Statistical power is relevant for judging the quality of empirical research literatures because, compared to lower powered studies, studies that are highly powered are more likely to (a) detect valid effects, (b) buffer the literature against false positives, and (c) produce findings that other researchers can replicate. In short, the more power a study has, the better positioned it is to provide precise knowledge and make robust contributions to the empirical literature.

All else being equal, we believe that journals that publish empirical studies based on highly powered designs should be regarded as more prestigious and credible scientific outlets than those that do not. As such, we introduce a new index, called the *N-pact Factor* (NF), that can be used to rank journals with respect to the statistical power of the empirical studies they publish. To illustrate the utility of the NF, we examine empirical studies published in six well-regarded journals in social and personality psychology. We demonstrate that, overall, the statistical power of studies published in these journals tends to be inadequate by conventional standards. Moreover, we show that there is considerable variation among journals; some journals tend to consistently publish higher power studies and have lower estimated false positive rates than others. And, importantly, we show that some journals, despite their comparatively high impact factors, publish studies that are greatly underpowered for scientific research in psychology.

Although we focus in this article on the power researchers choose for their studies as a means for evaluating the quality of empirical research, we wish to be clear from the outset that it is not our intent to argue that statistical power is the only indicator of research quality. There are many ingredients involved in high quality research. A high quality study, for example, should be capable of addressing a theoretically or practically important problem. It should involve reasonable and established methods for assessing the constructs of interest. The relationship between theory and measurement should be explicit and clear. And the data should be analyzed in a competent manner. We focus on statistical power over these other factors for at least three reasons. First, one of the fundamental ingredients in the calculation of statistical power, sample size or *N*, can be objectively coded with little interpretational ambiguity. Although researchers, reviewers, and editors may disagree on whether the research questions addressed in an article are important or interesting, there is little room for debate on whether the sample size used to answer those questions was 25 vs. 225. Second, although power is only relevant in the context of null hypothesis significance testing (NHST), NHST, despite its detractors, remains the dominant way in which psychological scientists go about making decisions regarding statistical hypotheses. As we discuss later, sample size, which varies perfectly with power for a given effect size, is a more robust way to conceptualize these problems and, as a result, we emphasize sample size and power in the current report. Finally, the use of underpowered designs fundamentally undermines the integrity of scientific research. As we explain in more detail below, many of the problems that currently confront the field (e.g., the file drawer problem, the replicability crisis) stem in part from inadequate attention to statistical power. By using statistical power as a means for evaluating the quality of research published in empirical journals, we hope to call greater attention to the role it plays in the credibility of empirical research.

In short, we focus on statistical power because it is a fundamental ingredient in high quality research. It is our hope that the NF will provide a way for committees, scholars, and consumers to evaluate the quality of empirical journals based on a criterion other than citation rate alone. More importantly, however, we hope that an explicit ranking of the quality of empirical journals—something similar to a *Consumer Reports* ranking of journals—will help create incentives for journals and editorial boards to value higher power research designs when those designs are viable.

### Why Is Statistical Power Important for Research Quality?

Statistical power refers to the probability that a study will be able to find a statistically significant result when, in fact, the null hypothesis is false. Why is power necessary for high quality research? In the sections that follow we discuss three problems that can emerge when empirical studies are not designed in a manner that is sensitive to statistical power.

### Limitation 1. Low Power Studies are Less Capable of Detecting True Relationships

The most obvious limitation of under-powered research is that such research lacks the ability to detect true effects. In many situations in scientific psychology it is not uncommon for researchers to design a study to test a theoretically compelling hypothesis, but to design the study in such a way that the research only has a 50% chance of correctly detecting the effect if, in fact, the effect exists [7]–[8]. The costs of engaging in under-powered research are substantial. From a scientific perspective, the field misses out on the opportunity to learn more about valid, but undetected, statistical relationships—relationships that might be critical for evaluating competing theories, laying the groundwork for a new substantive area, or advancing potential interventions.

There are also costs from a human factors perspective. Part of the thrill of science is conducting research for the purposes of solving problems, discovering new things, and rigorously evaluating alternative theoretical predictions. When young researchers attempt to build knowledge via a process that has an accuracy rate that is no better than a coin flip, research begins to resemble a gamble; the study either “works” (i.e., the researcher “gets lucky”) or “doesn't work.” And when studies do not work, the research process can be demoralizing and potentially career-altering for otherwise talented graduate students and assistant professors who are trying to build a track record of publications.

In contrast, when research is designed to have high power from the outset, the research is better positioned to produce useful knowledge. Moreover, when a well-powered study “fails” to find a significant result, even that so-called “failure” provides useful scientific knowledge [9]. On average, journals that publish studies based on larger sample sizes are more likely than those that do not to provide useful knowledge to the field.

### Limitation 2. Literatures Based on Underpowered Studies have a Greater Proportion of False Positive Findings

A common misconception concerning statistical power is that power is irrelevant once the results are in and the findings have been shown to be statistically significant. According to this logic, sample size and statistical power might be worth considering at the research design stage, but, once a significant result has been found, it is no longer meaningful to ask whether the study had the power to detect the finding. If a significant result was found, the study was capable, *ipso facto*, of detecting it. There is no point in wondering whether a study that “worked” had the power to do so, nor is there much point in faulting a study that “worked” by referencing its relative lack of power.

What is not recognized among many researchers, however, is that low power studies that “work” can dramatically increase the proportion of false positives in the broader literature. In other words, the Type I error rate in a *collection* of studies is not determined by the alpha rate (5%) alone. The false positive rate among published studies is also a function of the average power of those studies. As the power of the typical study decreases, the ratio of false positives to true positives increases [10], [11].

This point is best understood with respect to a concrete example. Assume that, in a specific area of research, the null hypothesis has an *a priori* probability of being correct about half the time. Assume that researchers conduct 100 studies. In 50 of those studies researchers are testing hypotheses that are true and in 50 of them researchers are testing hypotheses that are false. Given an alpha rate of 5%, this implies that, among the 50 studies in which the researchers are testing research hypotheses that are false, 2.5 of those will produce Type I errors on average (i.e., 50×.05 = 2.5).

Does this mean that fewer than 5% of published findings in a literature are false positives? Not exactly. If we assume that only statistically significant findings are published—a simplifying assumption that is not too far from the truth [12]—then the proportion of significant findings that are false positives in the literature is equal to the number of false positives relative to the total number of published significant results (i.e., the bottom row in Table 1). This latter quantity is a function not only of alpha but also of statistical power.

**Table 1:** Correct and Incorrect Conclusions in NHST.

	REALITY	
CONCLUSION	Null hypothesis is true	Research hypothesis is true
Null hypothesis is true	A. Correct rejection (probability = 1 – α)	C. Type II error (probability = β)
Research hypothesis is true	B. Type I error (probability = α)	D. Correct hit (probability = 1 - β)(power)

*Note*. In Null Hypothesis Significance Testing (NHST), the null hypothesis of no effect or no difference is either true (cells A and B) or false (cells C and D). When the null hypothesis is true (i.e., the left-hand column), it is possible for a researcher to make an incorrect decision by obtaining a significant result and rejecting the null hypothesis (cell B). The probability of this happening is equal to α and is set to 5%, by convention, to help minimize the false positive. When the null hypothesis is false (i.e., the right-hand column), the researcher can make a correct decision by obtaining a significant result (cell D). The probability of this happening is (1 – β), or the statistical power of the test. When the null hypothesis is false, one can make an inferential error by failing to obtain a significant result (cell C). This error rate is defined as beta (β) and is commonly referred to as Type II error.

To see how this plays out, let us assume that the statistical power of a typical study is 20%. In such a situation, approximately 10 of the 50 studies in which the null hypothesis is false will yield significant results (i.e., 50×.20). Thus, the number of false positives relative to the total number of published significant findings is 20% (i.e., false positives/(false positives + correct hits)  =  2.5/(2.5+10)). That is, one out of every five published finding will be a false positive.

Consider how things would play out if the statistical power of a typical study in a research area were 80% instead of 20%. In this case, 40 of the 50 studies in which the null hypothesis is false will produce significant results (50×.80). The number of false positives remains the same (5% of 50, or 2.5). Thus, the number of correct hits (40) relative to the total number of significant results (42.5) is much higher than before: 94%. The proportion of false positives (6%) in the literature slightly exceeds the nominal 5% alpha level, but, comparatively speaking, the literature is of much higher quality than in the previous example in which the statistical power of a typical study was 20%.

The important point here is that *there are fewer false positives in a literature composed of high power studies than a literature composed of low power studies*. Indeed, when the statistical power of studies in a literature is low, the proportion of false positives published in that literature can be surprisingly high [13]. In contrast to conventional wisdom, statistical power does not become irrelevant once the results of a given study are known. Statistical power is absolutely crucial for ensuring that the knowledge being produced by a field is accurate. As such, journals that publish studies based on higher power designs are less likely to publish false positives, on average.

### Limitation 3. Under-Powered Studies are Less Capable of Producing Replicable Findings

One of the defining features of the scientific method is replication. Scientists assume that, if a researcher reports an empirical finding, other researchers using similar methods will be able to reproduce it. Scientists acknowledge that there can be errors in this process (i.e., even correct observations may not be replicated for a variety of reasons, including unknown moderators), but scientists nonetheless consider replicable findings to be more credible than findings that have yet to be replicated (e.g., due to lack of trying or due to failures to replicate despite earnest attempts to do so).

Statistical power plays a crucial role in replicability. Assume that some variable, *Y*, truly differs between two groups, such that the population effect size is *d* = .50. Moreover, assume that a researcher seeks to test this difference by sampling 32 people from Group 1 and 32 people from Group 2. The power of this design to detect the effect of interest is approximately 50%. That is, the researcher has a 50-50 chance of correctly rejecting the null hypothesis.

Let us further assume that the researcher gets lucky and the effect in his or her sample happens to be statistically significant. How likely is it that this researcher or another researcher will be able to replicate the finding that *Y* differs significantly between the two groups? Many researchers intuitively base their judgments of replicability on the *p*-value from the significance test: If the *p*-value is small (e.g., *p*<.001), the effect should be replicable [14]. However, as has been noted by many methodologists [14], the replicability of a finding is dependent on the statistical power of the design and not the outcome of a significance test from any one study. If a second researcher uses the same design and sample size, theoretically, he or she also has a 50% chance of detecting the effect. (Fortunately, recent systematic replication efforts in social psychology have used sample sizes that are larger than those used in the original studies [15], .) The power of a design, in other words, is statistically independent of the outcome of any one study [17].

The implications of this are profound. If a researcher attempts to replicate a perfectly valid effect using a design that only has 50% power, then he or she only has a 50% chance of detecting the true effect. Moreover, the probability that any two studies on the topic (e.g., an original study and a follow up study) will both produce statistically significant results is only 25% (i.e., .50 × .50). This suggests that, regardless of the veracity of the theories being tested, a research domain based on underpowered studies is likely to be full of “failures to replicate” and “inconsistent findings” [18]. Without taking power seriously, the cause of failed replications is ambiguous. The failures to replicate could be due to studying small effects with underpowered designs or they could be due to the invalidity of the focal hypothesis.

Again, the costs of this problem have the potential to be substantial for the field. For young researchers who are attempting to build on existing findings in the literature, the failure to use a highly powered design can lead them to fail to replicate the basic effect they are attempting to build upon (assuming it is not a false positive; see Limitation # 2), potentially stifling their careers and job prospects. Moreover, failures to replicate published findings have the potential to undermine the public's trust in scientific research in psychology, as has been witnessed in recent years in psychology [19]. It is possible that the so-called *replication crisis* or *crisis of confidence* in psychology [20] is an artifact of a long history of researchers using under-powered designs—a state of affairs that is guaranteed to reduce the odds that published findings can be replicated.

Most educated people have the intuition that science is a cumulative and self-correcting enterprise, involving a healthy dose of false starts, complete misses, and unbridled ambiguity. But if an overwhelming proportion of published research findings are not replicable simply due to power issues (i.e., problems that are under the direct control of researchers and the editorial standards of the journals in which they publish) rather than the uncertainty inherent in human behavior, then people have little reason to take psychological science seriously. In short, compared to journals that publish lower powered studies, journals that publish higher power studies are more likely to produce findings that are replicable—a quality that should factor into the reputations of scientific journals. Journals that choose to publish under-powered studies are indirectly contributing to the crisis of confidence in psychology.

(As a side note, it should be noted that Gregory Francis has examined power problems, but from the other direction [21]. Although most scholars would likely question the quality of research literature that produces heterogeneous estimates of an effect based on low powered studies, Francis has astutely noted that the appearance of more replications than is expected on the basis of statistical power is an indicator of publication bias. Simply put, if the power of a typical study is 50% and 10 studies are conducted, the odds that all 10 studies will produce significant results is .50^10^ or about 1 in a 1000. Thus, if 10 out of 10 underpowered studies in an area (or in a single report) report significant results, then that is an incredible set of findings indeed. Schimmack [22] has explicitly created an index called the *Incredibility Index* that quantifies this particular property in research literatures. To be clear, it is not the case that a series of replicated findings *per se* is indicative of publication bias. If studies are adequately powered, it is quite reasonable for all of them to all produce statistically significant results. For example, at 90% power, we would expect 9/10 studies, on average, to be statistically significant. But at 50% power, we would not expect 9/10 studies to produce statistically significant findings.)

## Methods and Results

The aims of this article are to introduce a new metric for ranking journal quality based on the statistical power of studies published in a given journal and to use that metric to compare the power of studies published in some of the top journals in social/personality psychology. It has long been known that the sample size, and accordingly, the statistical power, of research studies in psychology is low [7]. Moreover, despite repeated calls for methodological reform, previous studies suggest that such calls have gone unheeded. For example, when Sedlmeier and Gigerenzer [8] surveyed the statistical power of studies published more than 20 years after Cohen's original critique, they found that the typical power of studies had not changed (see [24], [25] for further discussion). We believe that explicitly ranking journals based on power may help incentivize journals, and therefore researchers, to increase their sample sizes. At the very least, we hope such rankings will help make the issue of statistical power more salient in the evaluation of empirical research.

To illustrate the NF, we examine six of the top journals in social and personality psychology (i.e., Journal of Experimental Social Psychology [JESP], Journal of Personality [JP], Journal of Personality and Social Psychology [JPSP], Journal of Research in Personality [JRP], Personality and Social Psychology Bulletin [PSPB], and Psychological Science [PS])). Although our long-term goal is to evaluate journals against one another within a variety of subfields in psychology, we focus on social/personality psychology in this article for two reasons. First, as social and personality psychologists, we wanted our initial investigation into these issues to probe the domains with which we are most familiar. Second, because many contemporary debates and discussions regarding the “replicability crisis” concern research in social psychology (see [26], [13], [20]), we thought it would be particularly useful to begin by indexing the relative quality of journals in this area. We focused on these journals in particular because they are generally regarded as the top empirical journals in social/personality psychology in North America. We recognize that there are some important social/personality journals that were not included in this report (e.g., the European Journal of Social Psychology and the European Journal of Personality). We hope to include these and other journals in our future surveys. We report all journals we coded, with the exception of Social Psychological and Personality Science, which does not have a Thomson Reuters impact factor yet, and has only been in circulation since 2010.

We operationalize the NF for a journal in a given year as *the median sample size of the studies it publishes within that year*. Although we have organized our discussion up to this point around statistical power rather than sample size *per se*, we use the metric of sample size for the NF rather than power in this particular report for three reasons. First, and most importantly, the metric of sample size is intuitive and can be widely understood by researchers, administrators, and the lay public, regardless of differences in exposure to the technical issues related to statistical power. Second, as we explain later, *N* is a useful way of computing both statistical power and the precision of parameter estimates (e.g., confidence intervals). Thus, whether one is an advocate of NHST or not, sample size is a useful and meaningful metric. Finally, for a given effect size, *N* and statistical power are perfectly correlated. As such, for the purposes of evaluating journals *relative to one another*, the information contained in average sample sizes and average power estimates is identical. Although we use *N* as the metric for the NF, we nonetheless use this information to derive power estimates and false positive rate estimates for each of the journals under consideration.

The use of sample size as our primary metric for indexing the quality of empirical research, however, raises an important issue. Namely, if certain journals tend to publish research that naturally focuses on large effect sizes, then those journals would be at a disadvantage with respect to the NF. Although those journals might publish studies that are, in fact, highly powered, they will fare poorly on the NF because the studies they publish might not need large samples to detect large effects with high power.

This is a valid concern. We do not doubt that there are some labs within social and personality psychology that are investigating larger effects than others. The “many labs” project [27], for example, provides compelling evidence that effect size estimates for certain anchoring problems are large (*d* = 2.42) compared to effect size estimates for other issues (e.g., the association between imagined contact and prejudice; *d* = 0.13). The most pertinent question for the present purposes, however, is whether this state of affairs varies systematically across *journals* in social/personality psychology. The journals we are examining are relatively broad in their scope, focusing on a variety of questions of interest to social and personality psychologists. We do not have any reason to believe that some social/personality journals are more likely than others to specifically publish research in domains in which the effect sizes are intrinsically larger than others.

The most salient substantive distinction among these journals is whether they bill themselves as being relevant to social psychology (e.g., *JESP*), personality psychology (e.g., *JP*), or both (e.g., *PSPB*). Thus, one way to frame the problem is to ask whether prototypical studies in social psychology tend to examine effects that are larger than those in personality psychology, either in virtue of the population effect sizes themselves or via the methods that are used to study them (e.g., experimental vs. correlational; see [28]). A quantitative analysis of research in social and personality psychology by Richard, Bond, and Stokes-Zoota [29], however, suggests that published effect sizes are comparable across these subfields. Specifically, Richard and colleagues [29] analyzed data from over 25,000 social/personality studies of 8 million people and found that the average effect size was equivalent to a Pearson correlation of .21 (a Cohen's *d* of .43). Moreover, although there was substantial variation in the effect size estimates across studies (*SD* = .15), this variation was not moderated by subfield; the average effect size of situational effects (*r* = .22) was similar to that of person effects (*r* = .19) (see also [30] and [31]). These kinds of findings indicate that, although some research areas might be concerned with larger effects than other areas, there is little reason to believe that these differences vary systematically across the non-specialty journals in social/personality psychology.

For each journal and for each year, starting in 2006 and ending in 2010, we drew a random sample of 20% of published empirical articles. In total, we coded 1,934 studies that were distributed across 824 articles. Two coders recorded the sample size of each empirical study reported in those articles. In cases where the two coders differed in their estimates by more than *N* = 30, the first author examined the studies in question and resolved the discrepancy. We excluded meta-analytic studies because our intention was to capture the sample sizes used by researchers when they have the freedom to choose their sample sizes. In meta-analysis, those choices are not made by the meta-analyst, but by the authors of the primary studies. We also excluded simulation studies because the trade-offs involved in using larger versus smaller samples in such studies are trivial in most cases. The database and codes are available online as supplemental material at http://osf.io/7im3n.

We used the following rules to deal with non-prototypical studies: For twin studies, we used the number of twin pairs as the unit of analysis. For studies of couples, families, dyads, other groups, we used as the unit of analysis whichever unit of analysis the authors focused upon. In cases where there were multiple samples in a study, we separately recorded the sample size of each sample if those samples were analyzed separately; if they were analyzed together, we recorded instead the aggregate sample size. In studies on accuracy or ratings of targets, we treated the number of targets as the sample size of interest. In longitudinal studies we recorded the number of cases at the initial wave as the sample size; if, however, the analyses critically depended on at least two waves (e.g., the analysis of difference scores), we recorded the number of cases available at both waves. As a general rule of thumb, we used the number of cases initially sampled, even if cases were excluded for various reasons (e.g., malfunctioning equipment, failure to follow instructions). We made exceptions when the initial sample was a broad sweep (e.g., mass testing sessions in Introductory Psychology) to identify participants who met the study criteria.

We elected to code studies from *PS* because it is a premier journal for research in social and personality psychology. However, it is also a journal that cuts across subfields of psychology more broadly, also publishing research on visual cognition, neuroscience, and developmental psychology, for example. Therefore, we focused on *PS* studies that fell within the traditional purview of social/personality psychology. We classified studies as being relevant to social and personality psychology if they were explicitly concerned with topics commonly studied in these fields (e.g., stereotyping, individual differences, emotion and affect regulation, social cognition, interpersonal relationships).

Table 2 summarizes the NFs for the journals we studied, organized by year and journal. One of the first things to note is that the unweighted average of the NFs (i.e., the overall median sample size) is 104. Another thing to note is that, although some journals exhibit minor year-to-year variability in their NFs, for the most part, there is not a strong tendency for journals to be generally increasing or decreasing in the sample sizes of the studies they publish. A linear model suggests that the typical sample size across all six journals is increasing by, at best, about 1.2 cases per year. To put these numbers in historical context, it is helpful to consider some data reported by Reis and Stiller [32]. They coded the sample sizes of studies published in the 1968, 1978, and 1988 volumes of *JPSP*. To deal with skewness in sample sizes, they coded all studies with sample sizes greater than 999 as 999. Using this coding system, they found that the mean sample size of studies published in each of those years was 141, 158, and 200, respectively. When we used the same coding system with our data, we found that the mean sample size for *JPSP* across the years 2006 to 2010 was 140. These data seem to suggest that, although there was an upward trend for *JPSP* studies to use larger sample sizes from 1968 to 1988, that trend has apparently reversed. It seems reasonable to conclude, based on these data, that there has not been a trend for researchers or journals in social/personality psychology to be more attentive to sample size and statistical power.

**Table 2:** Median Sample Sizes for each Six Empirical Journals in Social/Personality Psychology.

	Year					
	2006	2007	2008	2009	2010	NF-5
*JP*	211	160	162	184.5	173	178.1
*JRP*	81	126	165	133	140	129
*PSPB*	112	86	89	96.5	89.5	94.6
*JPSP*	80	86.5	93	95	96	90.1
*JESP*	114	55.5	88	77	98	86.5
*PS*	67	91	51.5	78.5	76	72.8

*Note*. NF-5  =  5-year N-pact Factor. JP  =  Journal of Personality, JRP  =  Journal of Research in Personality, PSPB  =  Personality and Social Psychology Bulletin, JPSP  =  Journal of Personality and Social Psychology, JESP  =  Journal of Experimental Social Psychology, PS  =  Psychological Science (social/personality articles only).

One of the important features of these data is the variation among journals in the sample sizes used in published reports. For example, a typical study published in *JESP* in 2010 had a sample size of 98, whereas a typical study published in *JP* in 2010 had a sample size of 173. Indeed, the relative ranking of the six journals we studied was highly stable across years (see the top portion of Table 3). In other words, journals that tended to have higher NFs than other journals in one year also tended to have higher NFs than other journals in other years. This was true despite the fact that (a) the actual studies published in the journals—as well as the researchers who conducted those studies—varied from one year to the next and (b) the journals, during the time span studied, had no explicit policies concerning the minimum sample sizes required for research they publish.

**Table 3:** Correlations Among 1-year N-pact Factors, 5-year N-pact Factors, and Citation Impact Factors across the Six Journals Studied.

	2006	2007	2008	2009	2010	NF-5
2006	1.00					
2007	.61	1.00				
2008	.55	.79	1.00			
2009	.78	.95	.88	1.00		
2010	.75	.87	.94	.96	1.00	
NF-5	.82	.91	.90	.99	.98	1.00
IF	−.46	−.25	−.57	−.40	−.49	−.48

Note. The upper matrix represents the stability of rank ordering of journal's 1-year N-pact Factors (NFs) from 2006–2010. The lower rows represent the correlations among NFs in any one year and the NF-5 and the citation Impact Factors (IF) of journals.

Given that there are stable differences across journals in their NFs, we created a composite index—the 5-year N-pact Factor (NF-5)—to capture the average sample size for each journal across the 5-year span studied. This index was derived simply by averaging the annual NFs for each journal over the 5-year span we sampled; we did not weight any one year more highly than another. As shown in Table 3, the NF-5 scores are strongly correlated with the yearly level NFs (.82> *r*s>.99). Moreover, *JP* has the highest NF-5 (178) whereas *PS* has the lowest (73). We focus our remaining analyses on the NF-5.

### What is the Statistical Power of the Typical Study to Detect a Typical Effect Size?

Previously we argued that sample size is a valuable metric against which to evaluate journals because it is one of the critical ingredients in statistical power. And, importantly, statistical power is necessary for (a) being able to detect real effects accurately, (b) buffering against high false positive rates in the empirical literature, and (c) producing replicable findings. Given the importance of highly powered research for the integrity of psychological science, we next examined the statistical power of studies typically published in these journals.

Statistical power is a function of three ingredients: α, *N*, and the population effect size [23]. Because alpha is set to .05, by convention, we can estimate the statistical power for a typical study published in each journal using the typical *N*s (i.e., the NF-5) reported in Table 2 and assuming a variety of population effect sizes. Table 4 reports the estimated statistical power of the typical study published in each journal for population effect sizes corresponding to *r* = .10 (*d* = .20), *r* = .20 (*d* = .41), *r* = .30 (*d* = .63), *r* = .40 (*d* = .87), and *r* = .50 (*d* = 1.15), respectively.

**Table 4:** Statistical Power to Detect Various Population Effect Sizes across Journals.

	Population Effect Size (*r*)				
	.10	.20	.30	.40	.50
*JP*	.27	.77	.98	.99	.99
*JRP*	.20	.63	.94	.99	.99
*PSPB*	.16	.49	.84	.98	.99
*JPSP*	.16	.48	.83	.98	.99
*JESP*	.15	.46	.81	.97	.99
*PS*	.13	.40	.74	.95	.99
*JPSP:ASC*	.14	.43	.78	.96	.99
*JPSP:IRGP*	.16	.49	.84	.98	.99
*JPSP:PPID*	.20	.60	.92	.99	.99

*Note*. JP  =  Journal of Personality, JRP  =  Journal of Research in Personality, PSPB  =  Personality and Social Psychology Bulletin, JPSP  =  Journal of Personality and Social Psychology, JESP  =  Journal of Experimental Social Psychology, PS  =  Psychological Science (social/personality articles only), JPSP:ASC  =  Attitudes and Social Cognition section of JPSP, JPSP:IRGP  =  Interpersonal Relations and Group Processes section of JPSP, JPSP:PPID  =  Personality Processes and Individual Differences section of JPSP. Power values assume a two-tailed test with an alpha level of .05.

For the sake of discussion, we focus on the results for *r* = .20 (*d* = .41). We focus on this effect in particular because the Richard et al. [29] meta-analysis found that the typical effect size in social/personality psychology is *r* = .21 (*d* = .43). We recognize, however, that the decision to focus on the power to detect the average effect in social/personality rather than specific effects documented in each empirical report is potentially controversial. We will return to this issue in the Discussion. But, for now, we note that the alternative to this approach is to estimate the power of studies in a *post hoc* way by computing the effect sizes observed in each study and, using that information and the study's sample size, compute the power of the study to detect the effect that was found. This *post hoc* or “observed” approach to computing power has been criticized by methodologists because observed effect sizes and sample sizes tend to be strongly negatively correlated in research literatures [33], [34]. As a result, small-*N* studies that actually produce significant results tend to report larger effect sizes than comparable large-*N* studies, thereby biasing their observed power estimates upwards [34].

Our focus on the power of studies to detect effects of *r* = .20 helps to solve this problem because, in most research contexts, researchers do not have a precise expectation concerning the size of the effect. In such situations, the average effect size observed in a field (via meta-analysis) is a helpful way to ground those expectations [35]. Moreover, because we are focusing on averages and expectations rather than any one study in particular, it is reasonable to inquire about the statistical power of a typical study in a journal to detect a typical effect. Such an analysis is informative about the norms for the journals in question. In short, by focusing on a specific effect size (*r* = .20), we are attempting to answer a relatively basic, yet important, question: “What is the power of the typical study in each of these journals to detect the average effect reported in social-personality psychology?”

**Figure 1:**
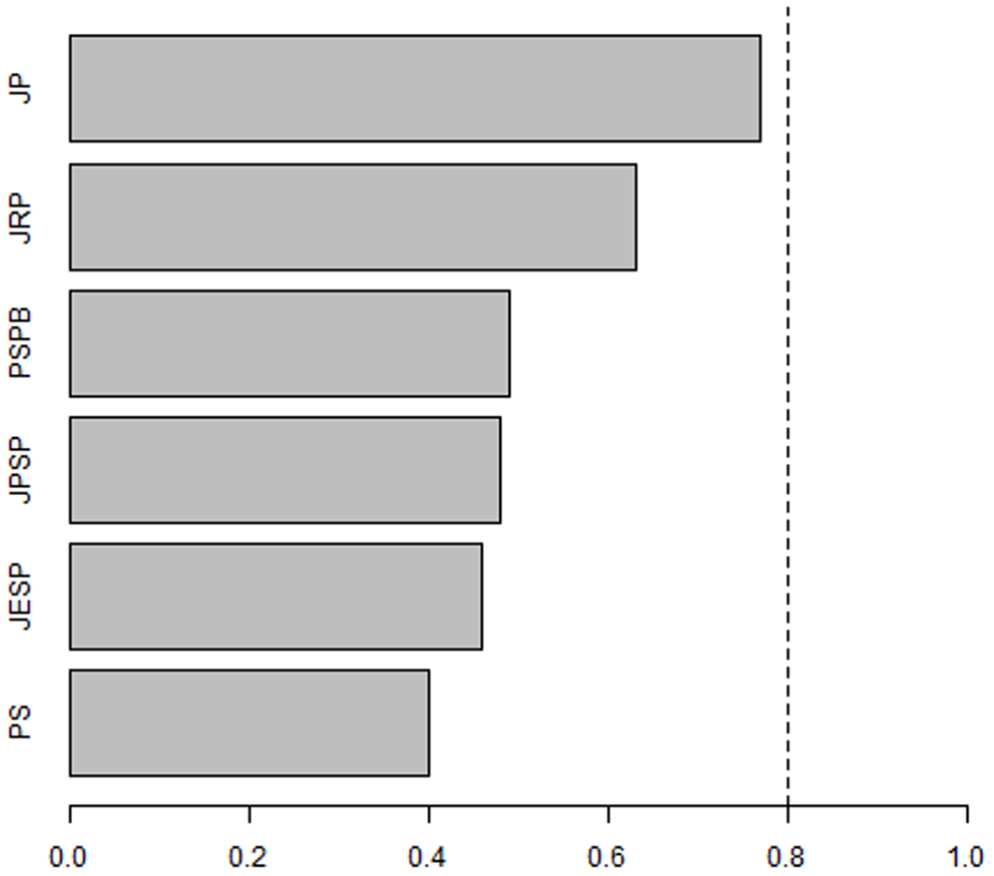
Rankings of Journals in Social-Personality Psychology with Respect to their Statistical Power. JP  =  Journal of Personality, JRP  =  Journal of Research in Personality, PSPB  =  Personality and Social Psychology Bulletin, JPSP  =  Journal of Personality and Social Psychology, JESP  =  Journal of Experimental Social Psychology, PS  =  Psychological Science (social/personality articles only). The hashed line represents the statistical power (80%) recommended by Cohen (1992).

Table 4 reveals that the typical study published in the social/personality journals we examined does not have adequate power to detect an effect equivalent to a correlation of .20. For example, *JPSP*, *PSPB*, and *JESP* each have close to or less than a 50-50 chance of correctly detecting population effects equivalent to a correlation of .20. This implies that, if, in fact, the null hypothesis is false and the population effect is equivalent to a Pearson correlation of .20, the typical study published in these journals is not any better able to detect that effect than a “test” based on tossing a coin. *JP* and *JRP* are exceptions to this trend. The typical study in *JRP*, for example, performed better than a coin flip (63% power) and the typical study published in *JP* approached what Cohen [23] considered an adequate degree of power (i.e., 80%), given the various tradeoffs involved in collecting psychological/behavioral data. The relative rankings of the journals with respect to the statistical power to detect a correlation of .20 are illustrated in Figure 1.

Given that the two journals explicitly focused on personality processes and individual differences (*JP* and *JRP*) had more power than the other journals, we decided to further explore potential subfield differences by separately examining studies published in the three sections of *JPSP*: Attitudes and Social Cognition (ASC), Interpersonal Relations and Group Processes (IRGP), and Personality Processes and Individual Differences (PPID). Averaging across years, the median sample size of studies published in these three sections was 79, 94.7, and 122, respectively. This corresponds to power values of .43, .49, and .60, respectively, to detect a population effect size of *r* = .20. Thus, there appears to be a tendency for research on personality and individual differences to utilize larger samples than research in social psychology and, accordingly, to have greater statistical power to detect the average effect sizes reported in social/personality psychology.

### What are the Estimated False Positive Rates of Findings Published in Journals?

Recall that most journals in psychology tend to publish articles for which the key findings were statistically significant [12]. Thus, with respect to Table 1, published articles are either false positives (Type I errors or rejections of the null hypothesis when it is, in fact, true) or Correct Hits (i.e., rejections of the null hypothesis when it is, in fact, false). The overall false positive rate in a literature can be defined as the number of false positives relative to the total number of significant effects (i.e., B/(B+D)), weighted by the relative *a priori* likelihood of null hypotheses being true or false in a research literature [10], [13] (see Table 1).

Table 5 explores the false positive rate estimated for various journals in social/personality psychology as a function of their power to detect an effect size equal to a Pearson correlation of .20. Because there is no way of knowing with certitude the *a priori* likelihood of the null hypothesis being true in various research literatures (i.e., *P*(H_0_)), for illustrative purposes we report false positive rate estimates under values of *P*(H_0_) of .50 and .80. A value of .50 corresponds to situations in which researchers are investigating hypotheses that, *a priori*, are just as likely to be true as they are to be false. This might be the case in situations in which the focal hypothesis predicts that Group A will score higher than Group B, but a credible alternative hypothesis predicts no effect or an effect in the opposite direction. According to our calculations, the false positive rate for journals in this scenario range between 6% and 11%. Among the higher power journals, such as *JP*, the false positive rate is close to the nominal alpha rate of 5%. The estimated false positive rate (11%) is more than twice the nominal alpha rate for *PS* under these assumptions.

**Table 5:** Estimated False Positive Rates of Findings Published across Journals, Assuming no Questionable Research Practices.

	*P*(H_0_) = true	
	.50	.80
*JP*	.06	.21
*JRP*	.07	.24
*PSPB*	.09	.29
*JPSP*	.09	.29
*JESP*	.10	.30
*PS*	.11	.33
*JPSP:ASC*	.10	.32
*JPSP:IRGP*	.09	.29
*JPSP:PPID*	.08	.25

*Note*. *P*(H_0_)  =  the *a priori* probability that the null hypothesis is true in a given research area. JP  =  Journal of Personality, JRP  =  Journal of Research in Personality, PSPB  =  Personality and Social Psychology Bulletin, JPSP  =  Journal of Personality and Social Psychology, JESP  =  Journal of Experimental Social Psychology, PS  =  Psychological Science (social/personality articles only), JPSP:ASC  =  Attitudes and Social Cognition section of JPSP, JPSP:IRGP  =  Interpersonal Relations and Group Processes section of JPSP, JPSP:PPID  =  Personality Processes and Individual Differences section of JPSP.

Table 5 also illustrates estimated false positive rates for each journal under the assumption that there is an 80% likelihood of the null hypothesis being correct, *a priori*. This assumption might characterize research areas in which investigators are testing risky or counter-intuitive hypotheses about how subtle experimental manipulations affect complex forms of behavior or areas of research in which there are multiple potential moderators of hypothesized effects, each of which has the potential to qualify or mask the focal effect being investigated. The estimated false positive rate under these circumstances is close to 28%. Indeed, according to our calculations, one in every three findings published in *PS* could be a false positive under these assumptions.

It is important to note that these estimates assume that researchers are *not* engaging in so-called *questionable research practices*, such as analyzing the data before the study is complete, selectively dropping dependent measures that “didn't work” but which would have been included if they had, etc. [36]. As Simmons and his colleagues note [36], such practices will inflate the false positive rate considerably. A survey by John, Loewenstein, and Prelec [37] revealed that questionable research practices are used with some degree of frequency in psychological research. If that is correct, then the numbers reported in Table 5 will underestimate the false positive rates of these journals considerably. The numbers we have reported are based exclusively on what is known about the average power of studies reported in these journals to detect typical effect sizes and reflect what the false positive rates would be if researchers did not engage in any questionable research practices.

### Do Journals with High Citation Impact Factors Publish Research with Higher N-Pact Factors?

**Figure 2:**
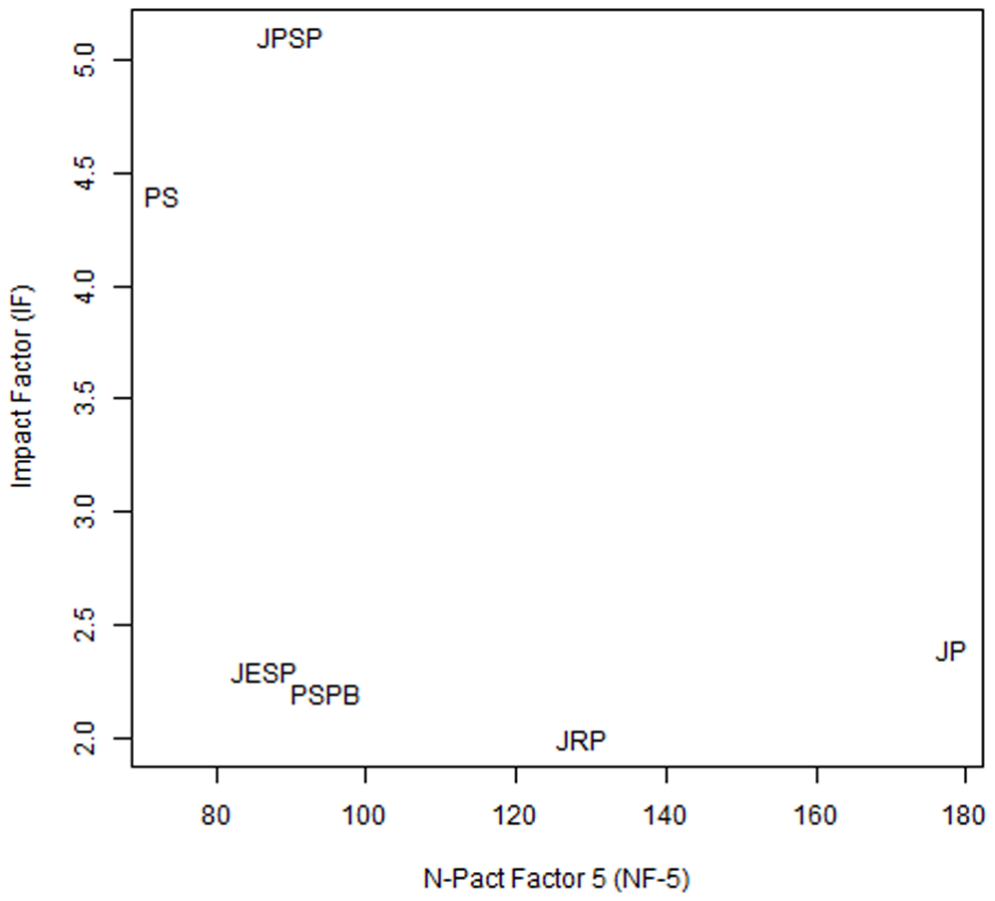
Journals plotted in a two-dimensional space defined by their 5-year N-Pact Factors (NF-5) and their citation Impact Factors.

Figure 2 illustrates the bivariate association between the 2011 Impact Factors of the journals we studied and their 5-year N-pact Factors. The figure reveals that the higher impact journals in social/personality psychology are not necessarily more likely to be publishing research based on high *N*, high power designs. In fact, the association between the NP and the 2011 IF is negative for every year sampled (see the lower row of Table 3). In other words, the journals that have the highest impact also tend to publish studies that have smaller samples.

Following suggestions from a reviewer, we also examined two alternative impact metrics, the eigenfactor (EF) and article influence (AI). The EF is a measure of a journal's “total importance to the scientific community”; the AI is a measure of the average influence of each of a journal's articles over the first five years after publication (see eigenfactor.org). Despite being based on different sources of information, these two metrics were highly correlated with one another among the 6 journals studied here (*r* = .96). Moreover, each was highly correlated with the Thompson Reuters Impact Factor (*r* = .94 and .98, respectively). They both correlated negatively with the NF-5: *r*s = −.68 and −.53, respectively.

## Discussion

The purpose of this article is to propose that one valuable way to conceptualize the “quality” of empirical studies and the journals that publish them is with respect to statistical power. Statistical power is critical for high quality research because studies that have higher power designs are more likely than those that do not to (a) produce precise estimates, (b) produce literatures with lower overall false positive rates; and (c) produce the kinds of replicable findings needed for a cumulative science. We have argued that journals that have a tendency to publish higher power studies should be held in higher regard than journals that publish lower powered studies—a quality we indexed using the N-pact Factor.

We used the NF to index the quality of research published in six well regarded journals in social and personality psychology (see Table 2). According to our analysis, the typical sample size used in studies published in our premier journals was 104. Some journals, however, tended to publish studies that substantially exceeded this value. The *Journal of Personality*, for example, had a NF-5 of 178 over the time span sampled here (i.e., 2006–2010). This indicates that the typical study published in the *Journal of Personality* has close to an 80% power to detect effects equal to a Pearson correlation of .20. The journal that we studied that had the lowest NF over the time span was *Psychological Science*. The typical social-personality study published in *PS* had 40% power to detect a correlation of .20.

For the most part, the relative ranking of the six journals we sampled was constant over the 5-year span, although some journals exhibited more year-to-year variability than others. There was not a general tendency for journals to increase in the typical sample size of the studies they published over time. We found that, in research areas where researchers might be testing risky or counter-intuitive hypotheses, the false positive rate could be as high as 25%. That is, one in every four published findings could be a Type I error. This estimate is a lower bound because it assumes that researchers are *not* engaging in questionable research practices. If they are (see John et al., 2011), then the false positive rate will substantially exceed 25%. We also found that journals which emphasized social psychology over personality psychology tended to have lower NFs. This was most clearly illustrated in considering the three sections of *JPSP*. The overall NF for the Attitudes and Social Cognition section was 79, whereas the overall NF for the Individual Differences and Personality Processes section was 122. This was the case even though previous meta-analyses indicate that the typical effect sizes examined in these subdisciplines are comparable [29].

### What about Research that is Naturally Focused on Larger Effect Sizes?

In this article we have focused on the statistical power of studies published in social/personality psychology to detect an effect equivalent to a Pearson correlation of .20. One potential concern with this decision is that certain areas of research might naturally require smaller sample sizes than others because they investigate population effect sizes that are substantially larger than *r* = .20. Although it does seem likely to us that some effects that psychologists study are larger than others and might require smaller sample sizes to accurately detect, we caution against this line of thinking for the purposes of evaluating the research quality of *journals* in social/personality psychology for two reasons.

First, as discussed previously, there is little reason to believe that effect sizes vary systematically across various journals in social/personality psychology. The journals we sampled publish research that spans a broad spectrum of topics in this discipline. And, to the extent that there are potential differences across journals with respect to content or methods, that line is probably best drawn between social psychology and personality psychology. But, as we noted previously, the meta-analysis by Richard et al. [29] found that most effect size estimates from research focused on situational factors and that focused on person factors are comparable. Such findings suggest that, although research in social and personality psychology may differ in a number of ways [28], the size of the effects upon which they focus is not one of them.

Second, although some researchers believe that the effects they are investigating are likely to be larger than *r* = .20, researcher's intuitions are often based on studies that “worked” rather than studies that did not. As a result, researchers' intuitions about the effect sizes in their areas are likely to be overestimates of the actual effects. When researchers toil in small-*N* fields for too long, they become accustomed to seeing large effect sizes because the only studies that can “work” in such situations are the ones that produce effect size estimates that are large enough to cross the *p*<.05 hurdle. This can happen, of course, if the actual effect sizes are large, but it can also happen incidentally by capitalizing on the sampling variation inherent in small-*N* studies [18]. Indeed, it is well known that effect size estimates are biased upwards in small-sample studies [34]. This bias can lead researcher's expectations for what a “typical” effect size is to be unrealistically large. As an analogy, if a person were to wear blinders that only allowed him to see objects that were at least 6 ft. tall, that person would likely grossly overestimate the average height of people in the population.

Third, with few exceptions, most hypotheses in social/personality psychology are framed as directional rather than quantitative predictions. For example, researchers might predict that, in light of certain theoretical considerations, Group 1 should have a higher mean on a certain outcome variable than Group 2, an interaction term should be greater than zero, or a correlation should be negative. It is rarely the case that a theory predicts something as precise as a .50 *SD* difference between groups, for example. Given that most predictions tested in psychology are of a directional variety where *any non-zero effect in a specific direction would be taken as being consistent with the theory*, it is reasonable to ask whether the typical study is capable of detecting relatively small, theory-consistent effects.

We think this suggestion is especially pertinent in light of recent debates about the robustness of well-known and highly publicized priming studies in social psychology. Based on the original publications of some of these controversial findings, it would seem that these effects are substantial—with effect sizes comparable to Cohen *d*s of .50 to 1.00 (i.e., Pearson correlations ranging from .24 to .45)(see [38]). Subsequent researchers, however, have had a difficult time replicating these effects (e.g., [39], [40]). Although some scholars have billed these failures to replicate as justification for questioning the whether these effects are “real,” a plausible alternative is that the actual effect sizes are much smaller than what has been previously assumed (see [41] for a discussion of this point). Our goal here is not to take a stance on this specific debate, but to call attention to two points. Namely, well-known and seemingly powerful findings in social/personality psychology may not have large effect sizes even if the canonical demonstrations of those findings reported large effect sizes (see [42]). Second, the debate over the existence of such effects might be more reasonably resolved if investigators calibrated their research designs against the expectation that, if specific effects are possible in certain experimental contexts, the effects are more likely to be small than large in size.

### Are High Power Studies Always Better?

Some researchers have expressed the concern that large sample sizes are a double edged sword. This apprehension stems from the fact that it is possible to obtain statistically significant results for even extremely small effects when sample sizes are large (see [43]). Although we agree that it is easier to detect small effects in large-*N* research (i.e., when power is high), we do not believe that this is a weakness of large sample research. In our view, this so-called problem is a limitation with theory testing in many areas of psychology. Meehl [44] noted decades ago that, when sample sizes are large in social science, the probability of empirically corroborating a directional prediction that has no validity whatsoever approaches 50%. Thus, finding empirical support for a typical prediction in psychological science is trivially easy once the problems of sampling error are eliminated. Meehl's point was not that statistical power makes hypothesis testing too easy; his point was that, if hypothesis testing seems too easy in highly powered situations, then that is a reflection of the imprecision of the hypothesis being tested. The solution is not to make the hurdle more challenging by using low-powered designs. The solution is to develop theories that make riskier (i.e., quantitatively more precise) predictions [45].

We appreciate the fact that many researchers have a disinterest in effect sizes below a certain threshold (e.g., −.10<*r*<.10) for *practical* reasons rather than theoretical ones. For example, if researchers are interested in how specific interventions might affect intergroup behavior, there may be a point at which the cost of the intervention exceeds the practical gains to be had. In such circumstances, researchers should make these thresholds clear *a priori* and design studies that are powerful enough to detect effects above the threshold at which they are invested [17]. For example, if researchers are only interested in effect sizes that are equivalent to correlations of .10 or larger, then they need sample sizes of at least 617 to have 80% power to detect the lower bound of this range. (We should be clear in noting that these thresholds should not be chosen on arbitrary grounds. Many researchers dismiss “small effects” without having a proper appreciation for just how theoretically and practically meaningful a so-called “small” effect can be [46], [47].) Furthermore, if researchers conduct a high-powered study and obtain a significant but too-small-to-be-interesting effect, they can identify it as practically unimportant and interpret it as such. Large samples in no way force researchers to interpret small effects as meaningful if they chose to discount effects within a certain range. To the contrary, large samples give researchers the level of precision necessary to determine whether an effect is practically meaningful.

One problem, however, is that not all research in psychology has obvious practical relevance. As Mook [48] famously noted in his defense of external invalidity, sometimes the point of research is simply to demonstrate that something is possible, under the right circumstances. In such situations, the magnitude of the effect may be less relevant for theory appraisal than, say, the question of whether the effect is in one direction vs. another. When this is the case, it is particularly important that researchers use highly powered designs because they need to insure that the design is actually capable of detecting an effect in the predicted direction, even if it is small. Without any constraints on what size effects would be practically meaningful, both small and large effects are relevant for hypothesis testing. Indeed, it has long been argued that small effects can be theoretically meaningful (e.g., [49]); thus, the integrity of research in basic science (i.e., research designed to test theoretical mechanisms rather than applications per se) hinges crucially on the ability of that research to detect small, theoretically consistent effects. Even if researchers consider the actual effect sizes to be somewhat arbitrary due to the vicissitudes of the way variables are manipulated and measured, estimating those effects well is necessary for building knowledge (e.g., reconciling contradictory findings), accurately testing theories, replicating and extending research, protecting the literature against false positives, and discovering the actual boundary conditions of certain effects.

### Limitations

The most important limitation of the NF is that no single metric can capture all important aspects of journal quality. No journal, and of course no researcher or body of research, should be judged solely on one aspect of research design. This is true of the IF and also of the NF. We do not intend for the NF to be the *only* index of journal quality. Rather, we believe that the NF complements the IF, and tracks some especially important components of research quality (i.e., precision, accuracy, and reliability)—components that are vital for good science, yet grossly undervalued in many areas of psychological science.

Another limitation of the NF is that, as it is currently quantified, it does not distinguish between within-subjects and between-subjects designs. Because the same total sample size provides much more statistical power for within- compared to between-subjects or mixed designs, our analyses underestimate the NF of journals that publish a higher proportion of purely within-subjects studies. However, our present analyses focus on research in social and personality psychology, and the primary question of interest in most social and personality research involves comparisons between people—people who vary in dispositional or attitudinal factors, people who differ in culture, and people assigned to different levels of experimental conditions. Nevertheless, before applying the NF to other subdisciplines (e.g., memory, visual cognition), it is important to calculate power appropriately for each type of design. Ultimately, this may require that the NF only be used to compare journals *within* specific fields (e.g., journals with social/personality psychology, journals within cognitive neuroscience) rather than across fields. That is, it is most defensible to use the NF to compare two journals in social psychology against one another than to compare a journal in social psychology with a journal in visual cognition.

Finally, we have discussed the value of sample size within a NHST framework. We have used this framework because the vast majority of psychologists use NHST as means of analyzing data and testing theories. We agree with critics of NHST, however, who have argued that not only is NHST a poor framework for theory testing (e.g., [45]), it has little to add above and beyond parameter estimation approaches. In parameter estimation approaches (e.g., [50]) the goal of research is to estimate parameters of interest, whether those are differences between conditions, patterns of means, or correlations. Indeed, once one begins thinking in terms of parameter estimation, the concept of statistical power becomes irrelevant [17]. Importantly, sample size is crucial in both NHST and parameter estimation approaches to empirical science and, as such, the *N*-pact Factor is a valuable way to rank journals regardless of one's preference. In an effect estimation paradigm, researchers will want to consider how precise they want their estimates to be. In early studies, for example, researchers might be more concerned with ballpark estimates and less concerned with getting the estimate exactly right. In situations where theory testing involves being able to rule out quantitatively similar predictions, however, getting tighter estimates has enormous value. An emphasis on effect sizes and precision of estimates is preferable to the dichotomous thinking of NHST for many reasons, perhaps most importantly because it encourages meta-analytic thinking–every finding is just more grist for the mill, and the larger your sample size the more grist you are contributing to the meta-analytic mill. We believe that the NF is useful whether one adopts the NHST framework, the parameter estimation framework, or even a Bayseian framework.

### Goals, Recommendations, and Future Directions

One of our goals is to encourage journals (and their editors, publishers, and societies that sponsor them) to pay attention to and strive to improve their NFs. By highlighting the role of sample size and power in high quality research and ranking journals with respect to their *N*-pact Factors, we hope to create reputational incentives for journals to increase their NF scores over the years. Another goal of our efforts is to provide people with an additional heuristic to use when deciding which journal to submit to, what to read, what to believe, or where to look to find studies to publicize.

One potentially useful future extension of the *N*-pact Factor is to consider the typical sample size or statistical power used by a given researcher. It is not uncommon for promotion committees, for example, to ask questions about the citation rates and impact of an individual researcher, as quantified, for example, with the *h*-index (where h is defined as the maximum number of papers a researcher has that have been cited at least *h* times). Like journal impact factors, such metrics emphasize something important that might not reflect the quality of the research itself. However, something that does (e.g., something based on sample sizes, power, or precision) could prove to be a valuable way to supplement traditional evaluations of the work that a researcher produces.

We close by noting that the goal of improving power and sample sizes in empirical studies in psychology is potentially in tension with other laudable goals. For example, multi-method designs, longitudinal designs, and ecologically valid designs are necessary for addressing many of the challenging questions in the field, but they are harder to implement using larger versus smaller sample sizes. Therefore, as researchers and journals aim to increase their power, they should also make sure to balance this goal with the goal of using rigorous methods and, when relevant, producing externally valid results. It would be lamentable if, in the pursuit of large sample sizes, journals began to publish a disproportionate number of studies based on Amazon Mechanical Turk workers or vignette studies of Introduction to Psychology students. These studies have their place, of course, but our preference would be for people to use whatever methods they believe are appropriate for the research question at hand and simply scale up the sample size in a way that increases the precision of the research.

Can we have it all? In short, no. It would be difficult for researchers in our field to continue publishing at current rates while also increasing their power. Something has to give. Like other people in our field (e.g., [51]), we believe that what has to give is the excessive rate of publication that has become the norm in social and personality psychology. As anyone on a hiring, award, or promotion committee lately has surely noticed, the expectations for number of publications have increased to the point where the most productive members of our field are publishing 20 or more articles per year. Some of this is due to increased collaboration, which is a positive development, but some of it is in response to the pressures and incentives in our field, which currently focus almost exclusively on quantity, often at the expense of quality. We hope that introducing the NF will help counteract this trend and create an incentive for journals, and therefore researchers, to value the ability of research to produce precise, accurate, and replicable research.

A “slow research movement” [51] would be a useful development for many reasons. First, rather than diluting their resources (e.g., running 10 studies with *N* = 50 each), researchers could focus their limited resources on one or two well-powered studies (e.g., running 2 studies with *N* = 250 each), which would provide more precise effect estimates and more robust knowledge. Second, a greater proportion of our published findings would be replicable. Third, if we all ran fewer but larger studies and published fewer but more informative papers, this would also help avert another impending crisis: the shortage of editors and reviewers to handle all of the manuscripts coming in (not to mention the shortage of readers to read the published articles).

### Summary

How should we gauge the quality of research journals in psychology? In this article we have argued that the average statistical power of studies published by journals provides one useful way of quantifying the quality of research published in those journals. All things being equal, studies based on large sample sizes are more likely to detect true effects, limit the number of false positive rates in the research literature, and produce replicable findings. Our analyses indicate that the N-pact Factor is a useful way of ranking well-regarded journals in social/personality psychology. It is our hope to continue ranking journals with respect to the NF in the years to come in order to incentivize competition among journals on something other than their impact factors alone.
